# Effect of Curcumin in Comparison with Trichostatin A on the Reactivation of *Estrogen Receptor Alpha* gene Expression, Cell Growth Inhibition and Apoptosis Induction in Hepatocellular Carcinoma Hepa 1-6 Cell lLine

**DOI:** 10.31557/APJCP.2020.21.4.1045

**Published:** 2020-04

**Authors:** Masumeh Sanaei, Fraidoon Kavoosi, Mehrnoosh Arabloo

**Affiliations:** 1 *Research Center for Non-Communicable Diseases, *; 2 *Student of Research Committee, Jahrom University of Medical Sciences, Jahrom, Iran. *

**Keywords:** Curcumin, Trichostatin A, Estrogen receptor alpha, Hepatocellular carcinoma

## Abstract

**Background:**

A multistep process with an accumulation of epigenetic alterations of tumor suppressor genes (TSGs) can induce cancer. Abnormal regional hypermethylation and histone deacetylation of several TSGs has been observed in hepatocellular carcinoma (HCC). Acetylation and deacetylation of histone are carried out by histone acetyltransferase (HAT) and histone deacetylase (HDAC) respectively. Besides, DNA methylation is carried out by DNA methyltransferases (DNMTs). Previously, we evaluated the effect of DNA demethylating agents and histone deacetylase inhibitors on HCC and colon cancer. This study aimed to evaluate the effect of curcumin (CUR) in comparison with trichostatin A (TSA) on *estrogen receptor alpha (ERα)* reactivation, apoptotic induction, and cell growth inhibition in HCC.

**Methods:**

the cells were cultured and treated with various concentrations of CUR and TSA and the MTT assay, flow cytometry assay and Real-Time RT-PCR were achieved to determine cell viability, cell apoptosis, and *ERα *gene expression respectively.

**Results:**

CUR indicated dose and time-dependent antiproliferative effects (P < 0.035). A similar antiproliferative effect was observed by TSA (P < 0.001). Both compounds indicated significant apoptotic effects in all different periods (P < 0.001), CUR indicated a more significant apoptotic effect than TSA (P < 0.001). The ERα gene expression quantity was increased significantly by treatment with CUR and TSA (P <0.012).

**Conclusion:**

CUR and TSA play important roles in restoring the ERα resulting in cell growth inhibition and apoptosis induction. Therefore, ERα may be a potential target for therapeutic intervention in the treatment of HCC.

## Introduction

The most primary malignancy of the liver is hepatocellular carcinoma (HCC) which is a leading cause of cancer-related death worldwide (Balogh et al., 2016). The most common risk factors of HCC are hepatitis B virus (HBV) and hepatitis C (HBC) virus infections (Zidan et al., 2012). Besides, associations between epigenetic alterations and cancer induction are well known. A multistep process with an accumulation of epigenetic alterations of tumor suppressor genes (TSGs) can induce cancer (Esteller, 2007). One of the main mechanisms of carcinogenesis is the hypermethylation of CpG islands in the promoter region of TSGs (Yang et al., 2017). Abnormal regional hypermethylation of the 5′ region of several TSGs has been observed in HCC (Baylin et al., 2011). Down-regulation of a variety of TSGs expression because of abnormal hypermethylation in their promoter regions could play a critical role in liver tumorigenesis (Nishida et al., 2011). DNA methylation is carried out by DNA methyltransferases (DNMTs) which transfer a methyl group to the cytosine of CpG dinucleotides. There is a family of these enzymes in the eukaryotes with five members, including Dnmt1, Dnmt2, Dnmt3a, Dnmt3b and Dnmt3L (Li., 2002; Bestor., 2000). DNMT1 is crucial for the maintenance of DNA methylation, which is important for the initiation of chromatin remodeling and regulation of gene expression (Espada et al., 2004; Milutinovic et al., 2004). More than 600 natural compounds have been reported with a pharmaceutical activity which many of them have anti-cancer activity, one of which is curcumin (CUR), diferuloylmethane, a powerful chemo-preventive, and anti-tumor agent (Bar-Sela et al., 2010). CUR exerts its effect through epigenetic modification by targeting numerous epigenetic factors such as DNMTs, histone acetyltransferase (HAT), histone deacetylase (HDAC) and miRNAs (Reuter et al., 2011; Balasubramanyam et al., 2004). Thus, the regulatory roles of CUR include DNMTs inhibition, histone modulations, and miRNA regulation (Boyanapalli et al., 2015). Furthermore, histone deacetylation is associated with carcinogenesis. Acetylation and deacetylation of histone carry out by HAT and HDAC respectively. In many tumors, the balance between HAT and HDAC activity is altered (Bannister et al., 2011). Histone deacetylase inhibitors (HDACIs) play an important role in cancer prevention and cancer treatment by promoting acetylation of histones and non-histone protein substrates. They can induce apoptosis and cell cycle arrest by acetylation of histone proteins. These compounds are classified into four classes, including hydroxamic acids, short-chain fatty acids, cyclic peptides, and synthetic benzamides (Lakshmaiah et al., 2014). Significant inhibitory and apoptotic effects of hydroxamic acid trichostatin A (TSA) on HCC HepG 2 have been reported (Shi et al., 2014). 

It has been reported that HDAC inhibitor TSA in combination with CUR has greater antiproliferative and apoptotic effects than either agent alone in breast cancer (Yan et al., 2013). 

*Estrogen receptor alpha (ERα)* is one of the TSGs that has recently attracted the attention of researchers and its role in various cancers has been reported. It is expressed in many tissues such as the breast and liver and is a very important choice for endocrine therapy (Halon et al., 2011; Kanda et al., 2014). Previously, we demonstrated that genistein (GE) and TSA can induce apoptosis by re-activation of *estrogen receptor alpha (ERα)* in the HCC Hep G2 cell line (Sanaei et al., 2017). However, to our researches, few studies have evaluated the effect of CUR and TSA on ERα reactivation, apoptotic induction and cell growth inhibition of human HCC Hepa 1-6 cell. Therefore, in the current study, we investigated the comparative effects of these compounds on the ERα gene expression, cell growth inhibition and apoptosis induction in this cell line. 

## Materials and Methods

*Cell Lines and cell culture*

Human hepatocellular carcinoma Hepa 1-6 cells were provided from the National Cell Bank of IranPasteur Institute and maintained in Dulbecco’s modified Eagle’s medium (DMEM) supplemented with 10% fetal bovine serum, 1% antibiotics, including streptomycin sulfate, penicillin G sodium (Sigma), and amphotericin B (Sigma) at 37°C in 5% CO_2_ to promote attachment. The Hepa 1-6 cells were cultured and treated with drugs (CUR and TSA) after they attached and reached > 80 % confluence. Both agents were purchased from Sigma-Aldrich (St. Louis, MO, USA) and dissolved in dimethyl sulfoxide (DMSO) to prepare a stock solution and then all test concentrations were prepared by diluting this solution. Other compounds, including trypsin-EDTA, fetal bovine serum (FBS), and phosphate-buffered saline (PBS) were purchased from Sigma Chemical Co. (St. Louis, MO, USA). Annexin-V-(FITC) and propidium iodide (PI) were prepared from sigma (Becton-Dickinson, San Diego, CA) too.

*Cell growth and cell viability assay*

Using MTT assay, the effect of CUR and TSA on the cell viability was measured. First, the cells were cultured into 96-well plates (at a density of 4 × 10^5^ cells per well) and allowed to adhere overnight. After 24 h of culture, the culture medium was removed and a medium containing different doses of CUR (0.5, 1, 5, 10 and 25 μM) and TSA (0.5, 1, 5, 10 and 25 μM) were added, except control groups incubated with DMSO only. After treatment times (24 and 48 h), the treated cells were washed twice with FBS, maintained in a medium containing MTT for 4 h and then the formazan crystals were dissolved in DMSO and the absorbance was measured at 570 nm. All experiments were repeated three times. 

*Cell apoptosis assay*

For apoptotic effect determination, the Hepa 1-6 cells were cultured in 24-well plates at a density of 4 × 10^5^ cells/well and incubated overnight before treating with drugs. After 24 h, the cells were treated with CUR (5 μM) and TSA (1 μM), Based on IC_50_ values, for different periods (24 and 48 h). After treatment times, the culture medium was removed and all the adherent cells were harvested by trypsinization, washed with cold PBS, and then the cells resuspended in Binding buffer (1x). Finally, Annexin-V-(FITC) and PI were used to stain the cells according to the protocol and the apoptotic cells were counted by FACScan flow cytometry (Becton Dickinson, Heidelberg, Germany).

*Real-time Quantitative Reverse Transcription Polymerase*

*Chain Reaction (qRT-PCR)*

To determine whether CUR and TSA could reactivate the *ERα* gene expression, qRT-PCR was performed. In this regard, the cells were cultured and treated with CUR and TSA (5 and 1μM respectively, based on IC_50_ values) for 24 and 48 h and total RNA from the cells was extracted using the RNeasy kit (Qiagen, Valencia, CA) according to the protocol and treated by RNasefree DNase (Qiagen) to eliminate the genomic DNA before cDNA synthesis. The concentration of RNA was determined using a BioPhotometer (Biowave II Germany). By using the RevertAid™ First Strand cDNA Synthesis Kit (Fermentas, K1622 for 100 reactions), total RNA (100 ng) was reverse-transcribed to complementary DNA (cDNA) according to the protocol. 

Real-time RT-PCR was performed by the Maxima SYBR Green RoxqPCR master mix kit (Fermentas). ERα primers were obtained from our previous work (Kim S-H et al., 2010) which their sequences are shown in Table1. GAPDH was used as an endogenous control.

Data were analyzed using the comparative Ct (ΔΔct) method, the relative expression level of *ERα* was calculated by determining a ratio between the amount of the gene and that of endogenous control.

## Results

*Effect of CUR and TSA on cell viability of Hepa 1-6 cells*

**Figure 1:**
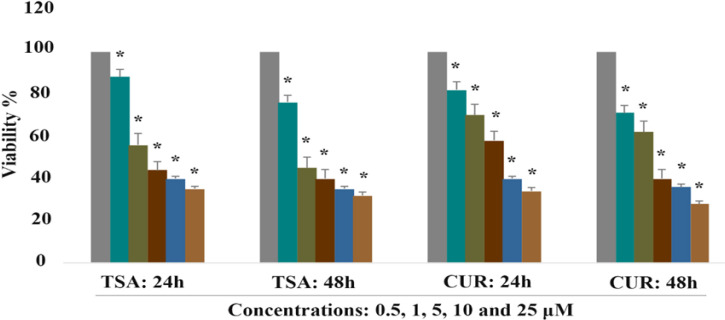
The Effect of CUR (0.5, 1, 5, 10 and 25 μM) and TSA (0.5, 1, 5, 10 and 25 μM) on the Hepa 1-6 Cell Viability. The effects were determined by the MTT assay. Data are presented as mean ± SD from at least triplicate wells and 3 independent experiments. Significant differences between treated cells and the control group are indicated by asterisks (*). The first column of each group belongs to the control group

The Hepa 1-6 cells were seeded in 96-well plates (at a density of 4 × 10^5^ per well) and treated with CUR (0.5, 1, 5, 10 and 25 μM) and TSA (0.5, 1, 5, 10 and 25 μM) for 1 and 2 days as mentioned above and then the cell viability was determined by MTT assay. As shown in Figure 1, CUR indicated dose- and time-dependent significant antiproliferative effects (P < 0.001). A similar antiproliferative effect was observed by TSA treatment as shown in Figure 1. (P < 0.002). The result of MTT assay demonstrated that CUR and TSA inhibited cell growth with an IC_50_ of ~5 and ~1μM respectively. 

*Effect of CUR and TSA on cell apoptosis*

**Figure 2:**
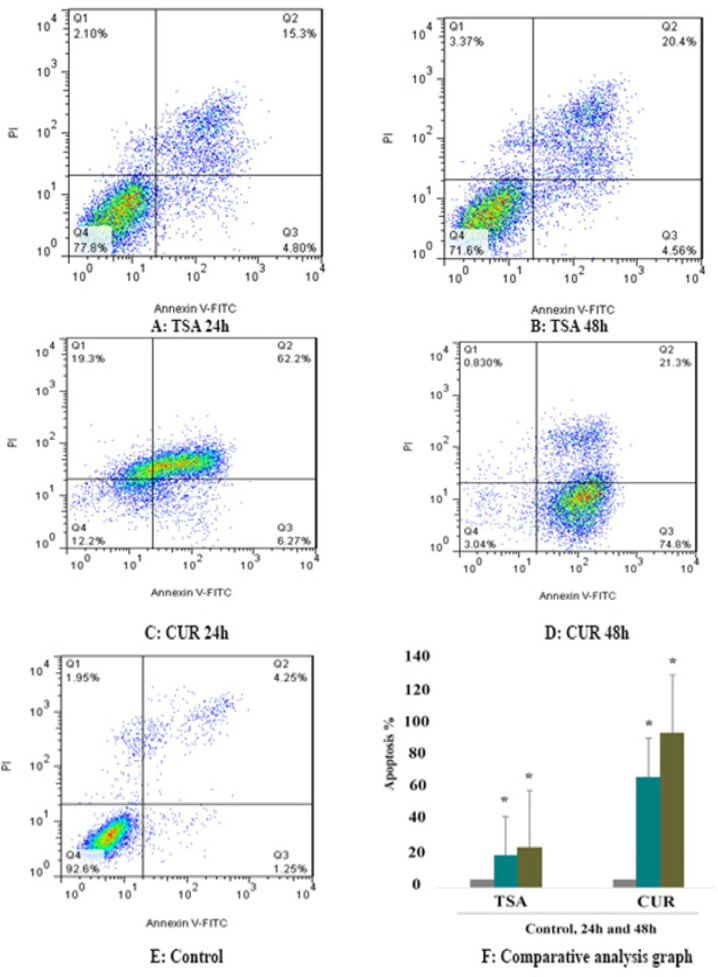
Apoptotic Effects of CUR and TSA on Hepa 1-6 Cell Line. Significant apoptosis was shown at different periods in a time-dependent manner (*P* < 0.001).

For determination of the apoptotic effects of CUR and TSA on Hepa 1-6, the cells were treated with CUR (5 Mμ) and TSA (1 Mμ) for 24 and 48 h and then the apoptotic effects of these compounds were determined by flow cytometry assay. The result of flow cytometry showed that both compounds indicated significant apoptotic effects in all different periods as shown in Figure 2 (P < 0.001). As seen in Figure 2, CUR indicated a more significant apoptotic effect than TSA (P < 0.001). The percentage of apoptotic cells is shown in Table 2.

*Effect of CUR and TSA on ERα gene expression*

**Figure 3:**
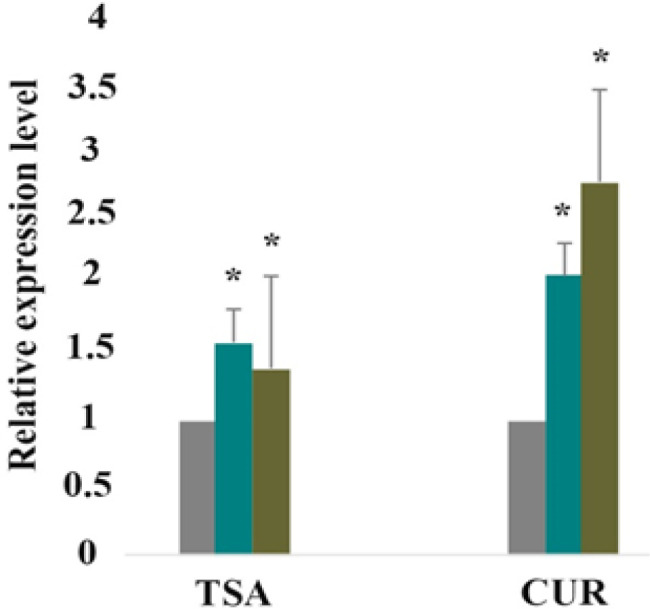
The Relative Expression Level of ERα. As can bee seen above, CUR and TSA increased ERα gene expression significantly. Data are presented as mean ± standard error of the mean from at least three different experiments. It should be noted that the effect of CUR was stronger than that of the TSA. Asterisks (*) indicate significant differences between treated cells and the control group

To determine the mechanism of CUR (5 Mμ) and TSA (1 Mμ) on ERα gene expression, RT-PCR was done to test the *ERα *gene expression quantity during treatment with these agents at different periods. As shown in Figure 3 and Table 3, the *ERα* gene expression quantity was increased significantly by treatment with CUR and TSA (P <0.012). The effect of CUR was more significant than that of the TSA. 

**Table 1:** Primer Sequence Used in the Study

Genes	Primer sequences
*ERα*	
Forward:	5ˈ-AGA CAT GAG AGC TGC CAA CC-3ˈ
Reverse:	5ˈ-GCC AGG CAC ATT CTA GAA GG-3ˈ

**Table 2:** Percentage of Apoptosis in the Groups Treated with CUR and TSA at Different Periods

Drug	Dose/ μM	Duration/ h	Apoptosis %	*P*-value
CUR	5	24	68.47	P < 0.001
	5	48	96.10	P < 0.001
TSA	1	24	20.10	P < 0.001
	1	48	24.96	P < 0.001

**Table 3:** Relative Expression Level of *ERα* Gene Expression

Gene	Drug	Dose (μM)	Duration (h)	Expression	*P*-value
*ERα*	TSA	1	24	1.6	0.020
*ERα*	TSA	1	48	2.4	0.001
*ERα*	CUR	5	24	2.1	0.001
*ERα*	CUR	5	48	2.8	0.001

## Discussion

Cancer cells are activated by genetic and epigenetic alterations, epigenetic processes such as hypermethylation and deacetylation of TSGs. These processes silence TSGs resulting in cancer induction. Hypermethylation creates a closed chromatin conformation that affects gene expression and involves cellular processes such as cell cycle, cell growth, cell differentiation, and cell apoptosis. Numerous medicinal plant-derived compounds have been reported to modulate the activation of diverse silenced genes in various cancers. Curcumin is a well-known anti-oxidative and anti-inflammatory agent which prevents several cancers via epigenetic regulation, including DNMTs inhibition, histone modifications, and microRNAs regulation (Boyanapalli et al., 2015). HDACs are also critical regulators of gene expression. Acetylation and deacetylation of histone proteins are achieved by two groups of enzymes, including HATs and HDACs. Histone deacetylation has been related to a broad range of cancer types. HADIs play an important role in cancer prevention and treatment. Histone deacetylase inhibitor TSA can restore silenced TSGs such as *ERα* by acetylation of these genes (Kim et al., 2010). In the present study, we showed that CUR and TSA can epigenetically inhibit cell growth and induce apoptosis in Hepa 1-6 cells and that CUR induces a more significant apoptotic effect than TSA. Besides, our work indicated that both compounds increase *ERα* gene expression significantly. The effect of CUR on *ERα* gene expression was more significant than the TSA.

Similar to our report, it has been shown that TSA can suppress the proliferation of primary hepatic cells and breast cancer cells by acetylation of histone (Kim et al., 2010; Xiong et al., 2012; Yang et al., 2000). There are many studies that have been reported that TSA inhibits growth and induces apoptosis in other cancers, including ovarian cancer, lung cancer epithelial-like cell (SKOV-3 and A549 respectively), urinary bladder cancer cells and esophageal squamous cell carcinoma (Hassan et al., 2014; Wang et al., 2017; Ma et al., 2015). In line with our result, it has been reported that CUR inhibits proliferation and induces apoptosis in HCC J5 cells (Cheng et al., 2008). The most evaluated component combinations are those that affect histone deacetylation (such as HDACIs) and DNA methylation (such as DNMTIs) (Ahuja et al., 2016; Azad et al., 2013; Egger et al., 2004). 

One of the mechanisms by which CUR exerts its mechanism is through epigenetic factors such as HDAC. Several studies have reported that CUR modulates various molecular pathways, including cell cycle proteins (e.g., cyclin D1) and apoptosis-related proteins (e.g., Bcl-2, caspases, DR, Fas) (Shishodia., 2013). CUR exerts an antiproliferative effect through DNMT1, it blocks the catalytic thiolate of C1226 of DNMT1 to exert its antiproliferative effect (Liu et al., 2009). Other signaling events inhibited by CUR include Jun/Ap-1 activation, phosphorylations catalyzed by protein kinases and prostaglandin biosynthesis (Nakamura et al., 2002; Shehzad et al., 2010; Koeberle et al., 2009). Our previous study indicated that TSA, as a histone deacetylase inhibitor, can up-regulate *ERα* gene expression and induce apoptosis in Hep G2 HCC (Sanaei et al., 2017). In the current study, we demonstrated that TSA induces apoptosis by the up-regulation of *ERα*. Similarly, this pathway has been reported for TSA in breast cancer (Stark et alo., 2013). Drug-induced accumulation of acetylated proteins such as p53, BCL6, and Hsp90 is one of the other mechanisms of suberoylanilide hydroxamic acid (SAHA) (Richon., 2006). The molecular mechanism of the apoptotic effect of SAHA in chronic myelocytic leukemia BV-173 cells is Bcr-Abl and c-Myc downregulation (Xu et al., 2005). It has been reported that TSA can activate certain hypermethylated genes, including *CDKN2B*, *CDKN2A*, *MLH1* and *TIMP3* in colon cancer cells after Dnmt1 inhibition by DNA demethylating agent such as 5-aza-dc (Cameron et al., 1999). which may support this thesis that CUR increases *ERα* gene expression by *DNMT1 *gene down-regulation. All reports that mentioned above are inconsistent with our results. Significant downregulation of *ERα* expression has been reported in breast cancer that is opposite to our result (Nejati-Koshki et al., 2014). Additionally, an in vitro study has reported that curcumin can inhibit colon cancer HCT-116 cell line as a dose dependent manner (Khameneh ZR et al., 2018). With regard to our report about the effects of *CUR* and *TSA* on *ERα* gene expression and other reports about the effect of CUR on DNMT1 inhibition, investigation of the effects of these compounds on *DNMT1* expression is recommended.

In conclusion, collectively, our result suggests an important role of CUR and TSA on ERα re-activation, cell growth inhibition and apoptosis induction of hepatocellular carcinoma, which may provide a new preventive and therapeutic strategy for cancer treatment.
